# Association of Breastfeeding Duration with 12-Month Postpartum Blood Lipids in a Predominately Lower-Income Hispanic Pregnancy Cohort in Los Angeles

**DOI:** 10.3390/ijerph19053008

**Published:** 2022-03-04

**Authors:** Zhongzheng Niu, Christine H. Naya, Lorena Reynaga, Claudia M. Toledo-Corral, Mark Johnson, Tingyu Yang, Brendan Grubbs, Nathana Lurvey, Deborah Lerner, Genevieve F. Dunton, Rima Habre, Carrie V. Breton, Theresa M. Bastain, Shohreh F. Farzan

**Affiliations:** 1 Department of Population and Public Health Sciences, Keck School of Medicine, University of Southern California, Los Angeles, CA 90039, USA; niuz@usc.edu (Z.N.); naya@usc.edu (C.H.N.); claudia.toledo-corral@csun.edu (C.M.T.-C.); markj939@usc.edu (M.J.); tingyuya@usc.edu (T.Y.); bgrubbs@usc.edu (B.G.); dunton@usc.edu (G.F.D.); habre@usc.edu (R.H.); breton@usc.edu (C.V.B.); bastain@usc.edu (T.M.B.); 2 Department of Health Sciences, College of Health and Human Development, California State University Northridge, Northridge, CA 91330, USA; lorena.reynaga.686@my.csun.edu; 3 Department of Obstetrics & Gynecology, Keck School of Medicine, University of Southern California, Los Angeles, CA 90039, USA; 4 Eisner Health, Los Angeles, CA 90015, USA; nlurvey@eisnerhealth.org (N.L.); dlerner@eisnerhealth.org (D.L.)

**Keywords:** breastfeeding, lipids, postpartum, longitudinal cohort, maternal health

## Abstract

Breastfeeding may protect women’s long-term cardiovascular health; however, breastfeeding-related postpartum lipid changes remain unclear. We aim to examine associations of breastfeeding duration with maternal lipids at 12 months postpartum. In a subsample (*n* = 79) of the Maternal and Developmental Risks from Environmental and Social Stressors (MADRES) cohort, breastfeeding status and duration at 3, 6, and 12 months postpartum were self-reported. Serum levels of lipids, including total cholesterol, triglycerides (TG), high-, low-, and very low-density lipoprotein cholesterol (HDL-C, LDL-C, VLDL-C), were measured from blood samples collected at 12 months postpartum. We used linear regression models to compare lipids by breastfeeding duration, adjusting for potential confounders. Women who were breastfeeding at 12 months had higher HDL-C (mean: 41.74 mg/dL, 95% CI: 37.27–46.74 vs. 35.11 mg/dL, 95% CI: 31.42–39.24), lower TG (80.45 mg/dL, 95% CI: 66.20–97.77 vs. 119.11 mg/dL, 95% CI: 98.36–144.25), and lower VLDL-C (16.31 mg/dL, 95% CI: 13.23, 20.12 vs. 23.09 mg/dL, 95% CI: 18.61–28.65) compared to women who breastfed for <6 months. No lipids were significantly different between women who breastfed for 6–11 months and for <6 months. Each month’s increase in breastfeeding duration was significantly, inversely associated with TG and VLDL-C and positively with HDL-C. Adjusting for fasting status, demographics, pre-pregnancy body mass index, breastfeeding frequency, and pregnancy complications did not appreciably change effect estimates. Breastfeeding at 12 months postpartum and a longer duration of breastfeeding in the first year postpartum were both associated with increased HDL-C and decreased TG and VLDL-C at 12 months postpartum.

## 1. Introduction

Cardiovascular disease (CVD) is the leading cause of death in women, accounting for one-third of all female deaths in the US in 2019 [1]. Long-term elevated levels of lipids, even moderately, can lead to CVD in later life [2]. Compared to men in the same age group, women generally have a less atherogenic lipid profile [3], as characterized by lower triglycerides and higher high-density lipoprotein cholesterol, because of the overall benefits of female hormones (e.g., estrogen) before menopause [4]. However, women also face various metabolic challenges during pregnancy when their hormone and lipid levels adapt to meet the fetus’s growing needs [5]. Lipid levels increase during pregnancy, reaching a peak in the third trimester [6]. After delivery, pregnancy-related lipid changes are thought to return to pre-pregnancy levels, but the contribution of breastfeeding practices, including duration, have not been explored in detail [7].

Breastfeeding has been suggested as a protective factor for women’s long-term CVD risk [8]. Epidemiological studies have associated breastfeeding with decreased long-term risk of Type 2 diabetes mellitus (T2DM), hypertension, and CVD in the decades after breastfeeding [9,10,11,12,13,14]. For instance, any breastfeeding occurrence, regardless of duration, has been associated with a lower risk of coronary heart diseases [10], stroke [11], and heart attack [12]. Moreover, the duration of breastfeeding, either cumulatively in the lifetime or averaged for each bearing child, is also important as longer breastfeeding duration has been associated with an even lower risk of metabolic conditions [9,13,14] and CVD [11,12] in several large epidemiological studies. These observations have led to the “reset hypothesis” that suggested breastfeeding as a critical player in restoring women’s pre-pregnancy weight, metabolism, and cardiovascular risk status [7].

However, breastfeeding-related lipid changes during the postpartum period remain unclear, and results from previous studies are inconsistent. A few previous studies found breastfeeding women had lower total cholesterol and triglyceride, and higher high-density lipoprotein cholesterol through 1 to 6 months postpartum compared to women who did not breastfeed, [15,16] although other studies found no significant differences [17]. Because physiological restoring is more profound and acute shortly after delivery (within 6 months), it may mask the role of breastfeeding on lipid changes in early postpartum, [18] and studies with a focus on lipids levels at a later postpartum time point may be more relevant to the association of breastfeeding with lipids. Besides, most of the previous studies were conducted 20–30 years ago when the breastfeeding rate was much lower than in recent years [15,16]. As more new mothers are following the WHO and American Academy of Pediatrics recommendations to breastfeed for a longer duration (e.g., 6 months or longer) [19,20], it is also critical to examine whether longer breastfeeding duration is associated with lipids beyond 6 months postpartum in a more recent cohort.

The association between breastfeeding and CVD risk factors, including lipids, is understudied among women from health disparity populations. Most of the previous studies were conducted among primarily non-Hispanic white populations, who have documented higher breastfeeding initiation rates and longer duration compared to women from underrepresented groups [21,22]. Fewer studies have focused on Hispanic women. The Maternal and Developmental Risks from Environmental and Social Stressors (MADRES) study is a prospective pregnancy cohort of predominantly Hispanic and socioeconomically disadvantaged women and children [23]. The present study aims to examine the association of longitudinally measured breastfeeding duration with lipid profiles at 12-months postpartum in a subsample of the MADRES cohort. We hypothesized that women with a longer duration of breastfeeding would have a less atherogenic lipid profile compared to women with a shorter duration of breastfeeding.

## 2. Method

### 2.1. Study Population

This study was nested within the Maternal and Developmental Risks from Environmental and Social Stressors (MADRES) study, an ongoing prospective pregnancy cohort. Details of the MADRES protocol have been described elsewhere [23]. Briefly, participants were recruited from Los Angeles County + USC (LAC + USC) Medical Center, the Women’s Health Center at Eisner Health, USC Obstetrics & Gynecology, and the South-Central Family Health Center. Inclusion criteria were: (1) <30 weeks pregnant at the time of enrollment, (2) ≥18 years of age, (3) singleton pregnancy, and (4) English or Spanish speaking. Exclusion criteria were: (1) HIV-positive status; (2) physical, mental, or cognitive disabilities that prevent participation; (3) current incarceration; or (4) multiple gestations. Maternal consent and HIPAA authorization for abstracting electronic medical records (EMR) were obtained prior to any study assessment. The Institutional Review Board at the University of Southern California approved all aspects of this study.

Participants were followed at multiple timepoints from the time of enrollment in pregnancy until 12-months postpartum by a combination of interviewer-administered in-person and telephone questionnaires in English or Spanish. Medical records were abstracted from all prenatal clinic visits to delivery. Anthropometric measurements of height and weight, as well as biospecimen collection, were conducted at in-person visits. At the time of this analysis, we included the first 87 women who had provided blood specimens and had lipids measurements at the 12-month postpartum timepoint. Participants who were pregnant again at the 12-month follow-up were excluded from the current study (*n* = 3). We further excluded five participants who had not provided any information regarding current breastfeeding practices as part of the 3-, 6-, and/or 12-month postpartum questionnaires. A total of 79 participants were included in the final analysis. A comparison between those included and excluded in the current analyses is shown in Supplemental Table S1. Using G*Power [24], we found our fixed sample size (*n* = 79) was sufficient to detect a small to medium effect (Cohen’s *f*^2^ > 0.1) with a power of 80% and the type I error (α) of 0.05.

### 2.2. Measurement of Breastfeeding

We prospectively obtained information on breastfeeding status and duration at 3-, 6-, and 12-months postpartum interviews using staff-administered questionnaires. At each time point, questions were asked as “Are you currently breastfeeding your baby?” and “How old was your baby when you completely stopped breastfeeding and/or pumping milk?”. For women who missed one or more postpartum interviews, we inferred breastfeeding duration and status where feasible. Specifically, if a woman reported no breastfeeding at an earlier timepoint and did not respond to a later interview, she would not be likely to reinitiate breastfeeding. Thus, her breastfeeding duration was recorded as the longest duration reported in the earlier interview. Similarly, if a woman reported breastfeeding at a later timepoint but missed earlier interviews, she would have been likely to breastfeed or express milk at an earlier time. If a woman reported a different duration of breastfeeding at different interviews, the reported duration at the later timepoint was used, as it likely would be more accurate than the report from an earlier time. If the reported month was missing, but the woman did report her breastfeeding status (i.e., yes/no) at one of the three timepoints, the breastfeeding duration should be longer than the latest month when she was currently breastfeeding. We combined all available information to determine the months of breastfeeding duration. We further categorized the duration of breastfeeding as “<6 months, 6–11 months, and currently breastfeeding at 12 months”. Mothers in the group “currently breastfeeding at 12 months” were actively milking at the time of blood drawn. Information on breastfeeding frequency (i.e., how many times of breastfeeding per day) was also collected at each follow-up visit (3-, 6-, and 12-months postpartum). We combined the longitudinal frequency data into one categorical variable with three levels: High-frequency (breastfeeding ≥ 7 times/day at 2 or 3 visits), Low-frequency (breastfeeding < 7 times at 1 or 2 visits), or None (0 at all visits). We chose 7 times/day to distinguish the high- vs. low-frequency groups, considering clinical recommendations and the declining trend of breastfeeding frequency over the postpartum period. [20,25] Participants were also asked if they were breastfeeding exclusively at each timepoint, which was not included in later analyses due to a large proportion of missingness.

### 2.3. Measurement of Blood Lipids

At the 12-month postpartum in-person follow-up, a trained study staff phlebotomist collected up to 50 mL of maternal blood using standard venipuncture protocols. Within 1 h of collection, samples were transported to the USC SCEHSC Integrative Health Core Molecular Biology Laboratory, processed, and stored at −80 °C until analysis. At the USC Metabolic Assay Core, an enzyme-linked immunosorbent assay (ELISA) was used to measure serum levels of lipids, including total cholesterol (TC), high-density lipoprotein cholesterol (HDL-C), low-density lipoprotein cholesterol (LDL-C), very low-density lipoprotein cholesterol (VLDL-C), and triglycerides (TG). Duration of time from last meal to venipuncture was recorded, and samples were categorized as fasting if participants reported at least eight hours from last meal to venipuncture.

### 2.4. Covariates

Using questionnaires, we collected information on maternal age, self-identification of race/ethnicity, marital status, education, annual household income, country of origin, obstetric history, and pre-pregnancy weight. We measured height at the first pregnancy study visit and further calculated pre-pregnancy body mass index (BMI) as self-reported pre-pregnancy weight (Kg) divided by the square of height (m^2^). Diagnoses of T2DM, pregnancy-related complications, including gestational diabetes (GDM), glucose intolerance, pre-eclampsia, hypertensive disorders of pregnancy, and depression, were abstracted from maternal medical records. Fasting status was ascertained at the time of blood draw and included as a covariate, as well as an effect modifier (see details below).

### 2.5. Statistical Analysis

We used frequencies and proportions to describe the distributions of categorical variables. For continuous variables, we examined histograms, skewness, and kurtosis to determine whether variables were normally distributed. Natural log-transformation was applied when variables were not normally distributed, which included all lipid measurements. Means and standard deviations were used to describe continuous variables. We analyzed the variance and used the F value to compare each lipid by categorical variables. We used Pearson correlation coefficients to assess the relationships between each lipid and continuous variables.

We fitted an unadjusted generalized linear regression model (Model 1) by regressing each lipid on breastfeeding duration categories. A set of potential confounders was selected based on a review of previous literature and analyses of the causal structure using directed acyclic graphs [26]. We adjusted these potential confounders in a sequential series of models. Model 2 adjusted for fasting status (<8 h vs. ≥8 h). Model 3 further adjusted for maternal age at study entry (year), ethnicity (Hispanic vs. non-Hispanic), country of origin (US, Latin America, Others), marital status (married, cohabitating, separated/unknown), annual household income (do not know or <$30,000 vs. ≥$30,000), and education (high school diploma or less vs. some college or above). The final Model 4 further adjusted for pre-pregnancy BMI (underweight or normal, overweight, obese) and order of the index pregnancy (first vs. second or later). To facilitate interpretation of effect estimates from these models where all lipids were natural log-transformed, we present the exponentiated marginal means of each lipid with 95% confidence intervals (CI), as this back-transformation produces numerical values that correspond to the measurement scales for interpretability. We calculated the *p-for-trend* by including the ordinal categories of breastfeeding duration as a continuous variable. We also used the continuous variable of months of breastfeeding duration as the independent variable to examine the association of each month’s increase of breastfeeding duration with lipids.

We further conducted several sensitivity analyses. Given the potential influence of pregnancy-related conditions on lipid levels, we further adjusted for T2DM, pregnancy-related complications, including GDM, glucose intolerance, pre-eclampsia, hypertensive disorders of pregnancy, and depression based on the final adjusted model (Model 4). To exclude the impact of active breastfeeding on the effect estimates of breastfeeding duration on lipids, we excluded those who were currently breastfeeding at 12 months when examining the association of each month’s increase of breastfeeding duration with lipids. To assess the influence of fasting status, we stratified the analysis by fasting status based on the final adjusted model. We examined the interaction of breastfeeding duration and breastfeeding frequency on lipids levels. Further, we ran models that concomitantly included breastfeeding duration and frequency to control the potential confounding effect of breastfeeding frequency.

## 3. Results

Characteristics in the whole sample and among the breastfeeding duration groups are present in Table 1. Overall, the participants were 29.62 (SD: 5.80) years old. More than 80% of the participants self-identified as Hispanic, and 44.30% were born in countries in Latin America. Most of the participants were married (29.11%) or cohabitating (45.57%), with an annual household income < $30,000 (65.82%), and with some college or above education (56.96%). The majority of participants were overweight (37.97%) or obese (27.85%) before pregnancy. About half of the participants were pregnant with their first baby at study entry (54.43%). Approximately half of the participants provided a fasting blood sample at the 12-month postpartum follow-up (51.90%). There were 25.32%, 17.72%, 12.66%, and 5.06% of the participants diagnosed with glucose-intolerance/GDM/T2DM, hypertensive disorder of pregnancy/pre-eclampsia, asthma, or depression, respectively. Of these 79 participants, 35.4% breastfed for <6 months, 30.4% for 6–11 months, and 34.2% were currently breastfeeding at 12 months. All of the aforementioned characteristics were generally comparable (*p* > 0.05) among the three breastfeeding duration categories, except that the proportion of obesity was marginally significantly (*p* = 0.05) higher among those who breastfed for <6 months (39.29%) compared to those who breastfed for 6–11 months (20.83%), or were currently breastfeeding at 12 months (22.22%).

Table 2 presents the exponentiated marginal means of each lipid with 95% CI by breastfeeding duration groups from Model 1 to Model 4. In the unadjusted model (Model 1), TG levels were significantly lower among those who were currently breastfeeding at 12 months (mean: 80.45 mg/dL, 95% CI: 66.20–97.77; *p* = 0.01) and slightly lower among those who breastfed for 6–11 months (mean: 112.58 mg/dL, 95% CI: 91.55–138.45, *p* = 0.69), compared to those who breastfed for <6 months (mean: 119.11 mg/dL, 95% CI: 98.36–144.25). The trend of decreasing levels of TG with a longer duration of breastfeeding was statistically significant (*p-for-trend* < 0.01). A similar pattern was observed for VLDL-C levels and the total cholesterol to HDL-C ratio. Conversely, HDL-C levels were significantly higher among those who were currently breastfeeding at 12 months (mean: 41.74 mg/dL, 95% CI: 37.27–46.74; *p* = 0.03) and slightly higher among those breastfed for 6–11 months (mean: 39.07 mg/dL, 95% CI: 34.65–44.05, *p* = 0.20), compared to those breastfed for <6 months (mean: 35.11 mg/dL, 95% CI: 31.42–39.24). The trend of increasing levels of HDL-C with a longer duration of breastfeeding was statistically significant (*p-for-trend* = 0.03). Breastfeeding duration did not appear to be related to total cholesterol levels or LDL cholesterol levels, as means did not differ significantly between breastfeeding duration categories. For all lipids, additional adjustment for fasting status (Model 2), maternal age and socioeconomic status (Model 3), and pre-pregnancy BMI and birth order (Model 4) had minimal influence on the observed marginal means. In sensitivity analyses, we further adjusted for T2DM, glucose intolerance, GDM, PE, HDP, and depression and found no meaningful changes in the marginal means from Model 4 (shown in Supplemental Table S2). We found no significant interaction effect of breastfeeding duration and frequency on lipids, and adjusting for breastfeeding frequency did not meaningfully change marginal means from Model 4 (shown in Supplemental Table S5).

Regression coefficients and 95% CI for the association of each month’s increase in breastfeeding duration with natural log-transformed lipids are present in Table 3. Results are consistent with findings from Table 2. With each month increase in breastfeeding duration, there were significant decreases in TG (*β* = −0.04, 95% CI: −0.07, −0.01), VLDL-C (*β* = −0.04, 95% CI: −0.07, −0.01) and the total to HDL cholesterol ratio (*β* = −0.02, 95% CI: −0.03, 0.00). Conversely, each month increase in breastfeeding duration was associated with increases of HDL-C (*β* = 0.02, 95% CI: 0.01, 0.04). No association was observed for months of breastfeeding duration with total cholesterol or LDL-C. Adjusting for potential confounders in Model 2 to 4 had minimal influence on effect estimates. In a sensitivity analysis, excluding women who were currently breastfeeding at 12 months had minimal influence on effect estimates, although some *p*-values increased, possibly due to a smaller sample size (Supplemental Table S3).

**Figure 1:**
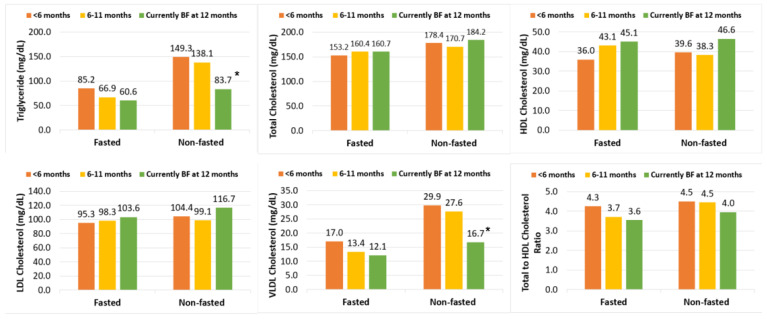
Exponentiated marginal means ^†^ of 12-month postpartum lipids by breastfeeding duration, stratified by fasting status. BF = breastfeeding. ^†^ Model estimates were back-transformed and presented as exponentiated marginal means for interpretability. * *p* < 0.05 compared to breastfed <6 months. Sample size (*n*) = 13, 14, 14 for breastfeeding ≥12 months, 6–12 months, and <6 months among non-fasted samples, respectively; Sample size (*n*) = 14, 10, 14 for breastfeeding ≥12 months, 6–12 months, and <6 months among fasted samples, respectively. All means were adjusted for maternal age, country of origin, marital status, household annual income, education, pre-pregnancy BMI and birth order.

To assess the potential impact of fasting status on the association of breastfeeding duration with lipids levels, we stratified the analyses by fasting status based on the final adjusted model (Model 4). As shown in Figure 1, TG and VLDL-C were higher among participants who did not fast than among those who fasted. The differences in TG, HDL-C, and VLDL-C were more evident among the non-fasting participants than among those fasted, but there was no significant interaction (*p* > 0.05).

## 4. Discussion

In a subsample of 79 women from a predominantly Hispanic and socioeconomically disadvantaged prospective cohort, we found that women who were still breastfeeding at 12 months postpartum had higher HDL-C and lower triglyceride and VLDL-C levels at 12 months postpartum compared to women who breastfed for <6 months. Consistently, each month increase in breastfeeding duration was associated with higher HDL-C and lower triglyceride and VLDL-C. These associations were robust after adjusting for potential confounders, including fasting status, demographics, pre-pregnancy BMI, parity, and pregnancy complications (e.g., GDM). These effect estimates remained the same after excluding women who were currently breastfeeding at 12 months. Overall, these results suggest that a longer duration of breastfeeding may be related to a less atherogenic risk profile, characterized by high HDL-C and low triglyceride and VLDL-C, at 12 months postpartum.

Traditionally, breastfeeding is recommended for its benefits to the infant [27], but the evidence is accumulating for its beneficial effect on the mother as well [28]. Recent systematic reviews suggest that promoting longer breastfeeding for more than 6 months is warranted for its clear benefits to the offspring and no clear harm to the mother [29]. In our predominantly Hispanic and socioeconomically-disadvantaged participants, 64.9% of mothers reported breastfeeding for at least 6 months, a prevalence similar to non-Hispanic white women (62.0%) participating in the US National Immunization Survey-Child (2016–2017) [19]. It is worth noting that the subsample in the current study was characterized by a high proportion (57%) of participants with some college or above education, although more than 60% of the participants reported annual household income <$30,000. This distinct characteristic indicated that the subsample would not represent the MADRES cohort or Hispanic and socioeconomically disadvantaged women at large (38.2% with some college or above education and 81.2% with income <$30,000, as shown in Supplemental Table S1). Nevertheless, in both the current subsample and the MADRES cohort (Supplemental Table S4), women who were foreign-born Hispanic appeared to be more likely to breastfeed for a longer duration, which could be due to reasons such as cultural, financial, social acceptance/pressures [30]. Further investigation is needed to identify and intervene on factors that can promote breastfeeding, particularly given the potential cardiovascular health benefits to mothers.

Our study found 12 months postpartum HDL-C was higher while triglycerides and VLDL-C were lower among those who were currently breastfeeding at 12 months compared to those who breastfed for <6 months. Previous studies have reported inconsistent findings on the association of breastfeeding with lipid levels, although most of the studies focused on earlier postpartum timepoints when post-pregnancy physiological changes may be more profound [18]. Our findings are consistent with two studies that also reported higher levels of HDL-C among breastfeeding women than non-breastfeeding women at 6 weeks and 4–12 weeks postpartum [15,16], but inconsistent with another study that found no differences in triglyceride and cholesterol levels (total, HDL-C, LDL-C) by breastfeeding status at 6–10 weeks postpartum [17]. Studies that measured lipids longitudinally suggested that breastfeeding may shift postpartum lipid trajectories. In a small longitudinal study of women (*n* = 34) who breastfed for at least 9 months, HDL, a subfraction of HDL-C, continuously increased in the first 9 months but decreased when women stopped breastfeeding [31]. On the other hand, triglyceride and VLDL-C levels decreased in the first 9 months and then increased at 12 months, and the trend was sharper among women who stopped breastfeeding after 9 months [31]. Physiologically, breastfeeding stimulates lipid mobilization from non-adipose tissues (e.g., the liver and muscles) into breast milk [32]. During breastfeeding, high prolactin levels stimulate lipoprotein lipase activity, which subsequently induces hepatic synthesis of VLDL, along with higher triglyceride clearance during lactogenesis [33,34]. Because fewer triglycerides of VLDL and LDL need to be exchanged with the HDL-C ester and VLDL surface remnant transferring increases, high serum levels of HDL-C are thus expected during breastfeeding [35]. It is worth noting that lipids were measured at the same time when some mothers were still breastfeeding in our study and previous articles. It is, therefore, important to understand whether breastfeeding has a differential influence on acute versus prolonged lipid levels.

Our findings suggested that the protective effect of longer breastfeeding duration on postpartum lipids may continue when women are no longer breastfeeding. We found that with each month increase of breastfeeding duration, there were significantly increased levels of HDL-C and decreased levels of triglycerides and VLDL-C. Excluding women who were currently actively breastfeeding at 12 months did not meaningfully change the effect estimates. These findings are consistent with previous retrospective studies that reported less atherogenic lipid profiles and reduced risks of cardiometabolic disease among women decades after breastfeeding (in their 50–60 years) [36,37,38,39]. On the contrary, in a prospective cohort study (Project Viva), when postpartum follow-up was extended to 3 years when all mothers had stopped breastfeeding, there were no significant differences in lipids by breastfeeding duration groups [40]. It is, therefore, critical for future prospective studies to better characterize postpartum lipids trajectories in women over a longer time and to compare these trajectories by breastfeeding duration.

We found the differences in HDL-C, VLDL, and triglyceride by breastfeeding were not significantly modified by fasting status, although overall triglyceride, total cholesterol, and VLDL-C levels were higher in non-fasting than in fasting samples. A large population-based Canadian cross-sectional study (*n* = 213,433) indicated no meaningful differences in HDL-C and total cholesterol by fasting duration, although LDL-C was increased up to 10% without fasting, and triglycerides were increased up to 20% [41]. Fasting is inconvenient for participants, especially for pregnant women and breastfeeding mothers, spurring an ongoing debate regarding the necessity of fasting in different settings (e.g., for purposes of screening, confirmatory testing, or research) [42]. In the literature, it is common that pregnancy and birth cohort study protocols show flexibility in the timing of the blood draw [40]. Valuable information may be lost by limiting to only fasting samples, particularly if similar trends may be observed with non-fasting samples. Future studies with a larger sample size are warranted to better examine the role of fasting status in the association of breastfeeding with lipids.

There are several limitations to our study. First, our study has a small sample size, limiting the power to detect differences in some lipids such as LDL-C. From power analyses, we found that our sample size can only detect the variation of lipids larger than 10%. Translating this variation to lipid levels accounts for approximately 20 mg/dL difference in total cholesterol or 15 mg/dL difference in triglyceride, which are clinically large differences. Second, about half of our participants did not provide fasting samples. Although we controlled for fasting status in multivariable models, caution is warranted when comparing our results to studies with fasting lipid levels. Third, our measures of breastfeeding focused on the duration of breastfeeding rather than exclusivity. Although exclusive breastfeeding plays an important role in infants’ health, it remains unclear whether supplementation would reduce the maternal impact of breastfeeding on postpartum lipid levels. In addition, the quantity, and regularity of milk production are also important in draining maternal energy repositories (e.g., glucose and lipids), but such information was not available in the current study. We found little evidence of an interactive effect of breastfeeding duration and frequency on lipids. Furthermore, there was no meaningful change in the effect of breastfeeding duration on lipids after adjusting for breastfeeding frequency, suggesting the robustness of breastfeeding duration’s effect on lipids. Future research with comprehensive measures of breastfeeding practices is needed to better understand the role of other breastfeeding practices in determining postpartum lipid levels. Fourth, residual confounding may exist, although we controlled for important confounders such as maternal demographics, pre-pregnancy BMI, and parity. Lipids levels before and during pregnancy may be important determinants of postpartum lipids levels, which were not available in the current study. Future studies with consecutive measures of lipids before, during, and after pregnancy are needed to better characterize the lipid trajectory and its relationship with breastfeeding.

In conclusion, we found that longer breastfeeding duration was associated with higher HDL-C and lower triglyceride and VLDL-C levels at 12-month postpartum among predominantly Hispanic and socioeconomically disadvantaged women. As an understudied sensitive window in women’s long-term cardiovascular health, pregnancy and the postpartum period warrant further investigations to identify potentially beneficial practices. While our sample size is limited and further studies are warranted, our findings begin to suggest that longer breastfeeding duration may have a cardioprotective by reducing potentially pro-atherogenic postpartum lipids levels in a health disparity population.

## Figures and Tables

**Table 1:** Sample characteristics by breastfeeding duration among 79 MADRES participants.

Characteristics	Overall (*n* = 79)	<6 Months (*n* = 28)	≥6–11 Months (*n* = 24)	≥12 Months (*n* = 27)	F/*χ*^2^	*p*
**Maternal age**	29.62 (5.80)	30.19 (5.56)	28.25 (5.18)	30.48 (6.64)	1.16	0.32
**Pre-Pregnancy BMI**	27.45 (4.99)	28.58 (6.23)	27.30 (3.29)	26.30 (4.85)	1.38	0.26
**GA at enrollment**	14.27 (6.54)	15.09 (6.95)	12.98 (5.80)	14.56 (6.77)	0.71	0.49
Maternal race *					6.63	0.36
*White*	73 (92.41)	25 (89.29)	24 (100.00)	24 (92.31)		
*Asian*	2 (2.53)	2 (7.14)	0 (0.00)	0 (0.00)		
*Black or African American*	1 (1.27)	0 (0.00)	0 (0.00)	1 (3.85)		
*More than one race*	2 (2.53)	1 (3.57)	0 (0.00)	1 (3.85)		
**Maternal country origin**					9.46	0.15
*Latin America*	35 (44.30)	10 (35.71)	16 (66.67)	9 (33.33)		
*USA*	37 (46.84)	16 (57.14)	7 (29.17)	14 (51.85)		
*Asia/Unknown*	7 (8.86)	2 (7.14)	1 (4.17)	4 (14.81)		
**Maternal ethnicity**					1.32	0.73
*Non-Hispanic*	15 (18.99)	7 (25.00)	3 (12.50)	5 (18.52)		
*Hispanic*	64 (81.01)	21 (75.00)	21 (87.50)	22 (81.48)		
**Maternal marital status**					4.52	0.61
*Married*	23 (29.11)	12 (42.86)	6 (25.00)	5 (18.52)		
*Cohabiting*	36 (45.57)	10 (35.71)	12 (50.00)	14 (51.85)		
*Separated/Unknown*	20 (25.32)	6 (21.43)	6 (25.00)	8 (29.63)		
**Annual household income**					6.02	0.11
*≥$30,000*	27 (34.18)	12 (42.86)	6 (25.00)	9 (33.33)		
*<$30,000*	52 (65.82)	16 (57.14)	18 (75.00)	18 (66.67)		
**Maternal education**					0.93	0.82
*Some college or above*	45 (56.96)	14 (50.00)	15 (62.50)	16 (59.26)		
*High school or lower*	34 (43.04)	14 (50.00)	9 (37.50)	11 (40.74)		
**Pre-Pregnancy BMI categories**					12.89	0.05
*Normal/Underweight*	27 (34.18)	10 (35.71)	8 (33.33)	9 (33.33)		
*Overweight*	30 (37.97)	7 (25.00)	11 (45.83)	12 (44.44)		
*Obese*	22 (27.85)	11 (39.29)	5 (20.83)	6 (22.22)		
**Birth order**					0.98	0.81
*First*	43 (54.43)	14 (50.00)	15 (62.50)	14 (51.85)		
*Second or later*	36 (45.57)	14 (50.00)	9 (37.50)	13 (48.15)		
**Fasting blood**					5.66	0.13
*Did not fast > 8 h*	41 (51.90)	14 (50.00)	14 (58.33)	13 (48.15)		
*Fasted > 8 h*	38 (48.10)	14 (50.00)	10 (41.67)	14 (51.85)		
**Lipid levels**						
*Triglyceride*	118.17 (66.98)	131.09 (58.32)	134.42 (81.35)	90.33 (53.34)	3.82	0.03
*Total Cholesterol*	168.39 (29.63)	165.40 (22.80)	169.93 (34.37)	170.12 (32.15)	0.22	0.81
*HDL Cholesterol*	40.19 (11.93)	36.94 (11.89)	40.24 (10.20)	43.52 (12.84)	2.16	0.12
*LDL Cholesterol*	104.56 (24.61)	102.25 (19.47)	102.81 (30.54)	108.53 (23.96)	0.53	0.59
*VLDL Cholesterol*	23.63 (13.40)	26.22 (11.66)	26.88 (16.27)	18.07 (10.67)	3.82	0.03
*Total to HDL Cholesterol Ratio*	4.52 (1.45)	4.94 (1.77)	4.44 (1.29)	4.15 (1.13)	2.12	0.13
**Pregnancy-related complications**						
**Glucose intolerant/GDM/T2DM**					4.22	0.24
*No*	59 (74.68)	19 (67.86)	17 (70.83)	23 (85.19)		
*Yes*	20 (25.32)	9 (32.14)	7 (29.17)	4 (14.81)		
**Hypertensive disorder of pregnancy/pre-eclampsia**					1.26	0.74
*No*	65 (82.28)	24 (85.71)	18 (75.00)	23 (85.19)		
*Yes*	14 (17.72)	4 (14.29)	6 (25.00)	4 (14.81)		
**Asthma**					2.42	0.49
*No*	69 (87.34)	26 (92.86)	21 (87.50)	22 (81.48)		
*Yes*	10 (12.66)	2 (7.14)	3 (12.50)	5 (18.52)		
**Depression**					0.77	0.86
*No*	75 (94.94)	27 (96.43)	23 (95.83)	25 (92.59)		
*Yes*	4 (5.06)	1 (3.57)	1 (4.17)	2 (7.41)		

Note: * There was 1 missing value not shown; MADRES = Maternal and Developmental Risks from Environmental and Social Stressors cohort; GA = gestational age (week); BMI = body mass index; GDM = gestational diabetes mellitus; T2DM = Type 2 diabetes mellitus.

**Table 2:** Exponentiated marginal means * of 12-month postpartum lipids by breastfeeding duration among 79 MADRES participants.

Breastfeeding Duration	Model 1		Model 2		Model 3		Model 4	
	Mean (95% CI)	*p* ^†^	Mean (95% CI)	*p* ^†^	Mean (95% CI)	*p* ^†^	Mean (95% CI)	*p* ^†^
**Triglyceride (mg/dL)**								
*12 months*	80.45 (66.20, 97.77)	0.01	80.86 (66.96, 97.66)	<0.01	79.30 (64.20, 97.96)	0.01	81.56 (66.13, 100.59)	0.01
*6–11 months*	112.58 (91.55, 138.45)	0.69	110.01 (89.98, 134.50)	0.56	99.11 (76.33, 128.69)	0.35	101.35 (78.33, 131.14)	0.37
*<6 months*	119.11 (98.36, 144.25)	Ref.	119.11 (98.97, 143.35)	Ref.	113.94 (91.23, 142.30)	Ref.	115.46 (93.06, 143.25)	Ref.
*P for trend*		<0.01		<0.01		0.01		0.01
**Total Cholesterol (mg/dL)**								
*≥12 months*	167.36 (156.78, 178.65)	0.66	167.61 (157.27, 178.64)	0.62	170.57 (158.55, 183.49)	0.57	172.96 (160.94, 185.88)	0.42
*≥6–12 months*	166.84 (155.68, 178.80)	0.71	165.70 (154.83, 177.33)	0.82	166.00 (151.67, 181.67)	1.00	167.99 (153.76, 183.54)	0.87
*<6 months*	163.96 (153.78, 174.82)	Ref.	163.96 (154.02, 174.55)	Ref.	165.97 (153.70, 179.22)	Ref.	166.59 (154.69, 179.40)	Ref.
*P for trend*		0.65		0.62		0.56		0.41
**HDL Cholesterol (mg/dL)**								
*≥12 months*	41.74 (37.27, 46.74)	0.03	41.75 (37.25, 46.78)	0.03	45.17 (40.47, 50.43)	0.02	45.02 (40.30, 50.30)	0.02
*≥6–12 months*	39.07 (34.65, 44.05)	0.20	39.03 (34.57, 44.06)	0.21	41.44 (36.18, 47.48)	0.26	41.51 (36.23, 47.57)	0.21
*<6 months*	35.11 (31.42, 39.24)	Ref.	35.11 (31.40, 39.27)	Ref.	37.98 (33.83, 42.64)	Ref.	37.67 (33.62, 42.22)	Ref.
*P for trend*		0.03		0.03		0.02		0.02
**LDL Cholesterol (mg/dL)**								
*≥12 months*	105.96 (96.97, 115.79)	0.40	106.09 (97.11, 115.90)	0.39	105.81 (95.31, 117.46)	0.43	107.78 (97.23, 119.48)	0.32
*≥6–12 months*	98.88 (90.00, 108.63)	0.80	98.34 (89.50, 108.05)	0.73	98.07 (86.20, 111.58)	0.76	99.55 (87.71, 112.98)	0.86
*<6 months*	100.54 (92.15, 109.69)	Ref.	100.54 (92.18, 109.66)	Ref.	100.27 (89.84, 111.91)	Ref.	100.85 (90.71, 112.12)	Ref.
*P for trend*		0.41		0.40		0.42		0.31
**VLDL Cholesterol (mg/dL)**								
*≥12 months*	16.09 (13.24, 19.56)	0.01	16.17 (13.39, 19.53)	<0.01	15.86 (12.84, 19.59)	0.01	16.31 (13.23, 20.12)	0.01
*≥6–12 months*	22.52 (18.31, 27.69)	0.69	22.00 (18.00, 26.90)	0.56	19.82 (15.27, 25.74)	0.35	20.27 (15.66, 26.23)	0.37
*<6 months*	23.82 (19.67, 28.85)	Ref.	23.82 (19.79, 28.67)	Ref.	22.79 (18.25, 28.46)	Ref.	23.09 (18.61, 28.65)	Ref.
*P for trend*		<0.01		<0.01		0.01		0.01
**Total to HDL Cholesterol Ratio**								
*≥12 months*	4.01 (3.58, 4.49)	0.06	4.02 (3.58, 4.50)	0.06	3.78 (3.35, 4.26)	0.06	3.84 (3.43, 4.30)	0.06
*≥6–12 months*	4.27 (3.78, 4.82)	0.28	4.25 (3.76, 4.79)	0.25	4.01 (3.45, 4.65)	0.30	4.05 (3.52, 4.65)	0.26
*<6 months*	4.67 (4.18, 5.22)	Ref.	4.67 (4.18, 5.22)	Ref.	4.37 (3.85, 4.96)	Ref.	4.42 (3.94, 4.97)	Ref.
*P for trend*		0.06		0.06		0.06		0.06

Note: Sample size (*n*) = 28, 24, 27 for breastfeeding ≥ 12 months, 6–12 months, and <6 months among non-fasted samples, respectively. Model 1 is unadjusted; Model 2 adjusted for fasting status; Model 3 further adjusted for demographic variables including maternal age, country of origin, marital status, annual household income, and education; Model 4 further adjusted for maternal pre-pregnancy BMI and birth order. * Model estimates were back-transformed and presented as exponentiated marginal means for interpretability. † *p* values indicate the significance of comparison with breastfeeding < 6 months as the reference group.

**Table 3:** Regression coefficients (95% confidence interval) for the association of each month increase in breastfeeding duration with natural log-transformed lipids at 12-months postpartum.

Natural Log-Transformed Lipids	Model 1		Model 2		Model 3		Model 4	
	Beta (95% CI)	*p*	Beta (95% CI)	*p*	Beta (95% CI)	*p*	Beta (95% CI)	*p*
**Triglyceride**	−0.04 (−0.07, −0.01)	<0.01	−0.04 (−0.07, −0.01)	<0.01	−0.04 (−0.06, −0.01)	<0.01	−0.03 (−0.06, −0.01)	0.01
**Total Cholesterol**	0.00 (−0.01, 0.01)	0.46	0.00 (−0.01, 0.01)	0.45	0.00 (−0.01, 0.01)	0.47	0.00 (0.00, 0.01)	0.29
**HDL Cholesterol**	0.02 (0.01, 0.04)	<0.01	0.02 (0.01, 0.04)	<0.01	0.02 (0.01, 0.04)	<0.01	0.02 (0.01, 0.03)	<0.01
**LDL Cholesterol**	0.01 (−0.01, 0.02)	0.36	0.01 (−0.01, 0.02)	0.36	0.01 (−0.01, 0.02)	0.42	0.01 (−0.01, 0.02)	0.25
**VLDL Cholesterol**	−0.04 (−0.07, −0.01)	<0.01	−0.04 (−0.07, −0.01)	<0.01	−0.04 (−0.06, −0.01)	<0.01	−0.03 (−0.06, −0.01)	0.01
**Total to HDL Cholesterol Ratio**	−0.02 (−0.03, 0.00)	0.02	−0.02 (−0.03, 0.00)	0.02	−0.02 (−0.03, 0.00)	0.02	−0.02 (−0.03, 0.00)	0.03

Note: Model 1 is unadjusted; Model 2 adjusted for fasting status; Model 3 further adjusted for maternal age, country of origin, marital status, annual household income, and education; Model 4 further adjusted for maternal pre-pregnancy BMI and birth order.

## Data Availability

The data underlying this article cannot be shared publicly due to the privacy of individuals that participated in the study. The data will be shared on reasonable request to the corresponding author.

## Supplementary Materials

The following supporting information can be downloaded at: https://www.mdpi.com/article/10.3390/ijerph19053008/s1. Table S1: Comparison of MADRES population characteristics between those included and excluded in the current analyses; Table S2: Exponentiated marginal means of 12-month postpartum lipids by breastfeeding duration among 79 MADRES pilot participants additional adjusting for pregnancy complications; Table S3: Regression coefficients (95% confidence interval) for the association of each month increase in breastfeeding duration with natural log-transformed 12-month postpartum lipids, excluding 27 participants who were currently breastfeeding at 12 months; Table S4: Comparison of MADRES population characteristics by breastfeeding duration categories; Table S5: Adjusted lipids levels by breastfeeding duration and frequency.
