# Supplementary material for: Association of Breastfeeding Duration with 12-Month Postpartum Blood Lipids in a Predominately Lower-Income Hispanic Pregnancy Cohort in Los Angeles
Source: Int J Environ Res Public Health. 2022 Mar 4;19(5):3008. doi: 10.3390/ijerph19053008 (PMC8910591; doi:10.3390/ijerph19053008)
Supplement: Supplementary file 1 [file ijerph-19-03008-s001.zip › ijerph-1530367-supplementary.pdf]

SUPPLEMENTAL FILES

Supplemental Table S1. Comparison of MADRES population characteristics between those included and excluded in the current analyses

		Excluded N=642	Included N=79	F/χ^2	P
**Maternal age**		28.13 (5.97)	29.62 (5.80)	2.09	0.04
**Pre-Pregnancy BMI**		28.70 (6.84)	27.45 (4.99)	2.01	0.05
**Maternal country origin**					
	Latin America	218 (33.96)	35 (44.30)	7.38	0.03
	North America	290 (45.17)	37 (46.84)		
	Asia/Unknown	134 (20.87)	7 (8.86)		
**Maternal ethnicity**				0.45	0.50
	Non-Hispanic	133 (22.32)	15 (18.99)		
	Hispanic	463 (77.68)	64 (81.01)		
**Maternal marital status**					
	Married	161 (25.08)	23 (29.11)	11.45	<0.01
	Cohabiting	196 (30.53)	36 (45.57)		
	Separated/Unknown	285 (44.39)	20 (25.32)		
**Household annual income**					
	≥$30,000	121 (18.85)	27 (34.18)	10.13	<0.01
	<$30,000	521 (81.15)	52 (65.82)		
**Maternal education**					
	Some college or above	245 (38.16)	45 (56.96)	10.34	<0.01
	High school or lower	397 (61.84)	34 (43.04)		
**Pre-Pregnancy BMI categories**					
	Normal/Underweight	217 (33.80)	27 (34.18)	3.37	0.19
	Overweight	188 (29.28)	30 (37.97)		
	Obese	237 (36.92)	22 (27.85)		
**Birth order**					
	First	460 (71.65)	43 (54.43)	9.89	<0.01
	Second or later	182 (28.35)	36 (45.57)		
**Glucose intolerant/GDM/T2DM**					
	No	430 (66.98)	59 (74.68)	1.91	0.17
	Yes	212 (33.02)	20 (25.32)		
**Asthma**					
	No	450 (70.09)	69 (87.34)	1.06	0.30
	Yes	94 (14.64)	10 (12.66)		
**PE/HTN/PIH**					
	No	506 (78.82)	65 (82.28)	0.51	0.47
	Yes	136 (21.18)	14 (17.72)		
**Depression**				2.34	0.13
	No	67 (10.52)	4 (5.06)		
	Yes	570 (89.48)	75 (94.94)		

Supplemental Table S2. Exponentiated marginal means* of 12-month postpartum lipids by breastfeeding duration among 79 MADRES pilot participants additional adjusting for pregnancy complications

Biomarkers	Breastfeeding duration	Marginal mean (95% CI)	P value[†]
**Triglyceride**			
	≥12 months (N=27)	**80.69 (57.29, 113.66)**	**0.03**
	≥6-12 months (N=24)	98.75 (67.61, 144.24)	0.50
	<6 months (N=28)	108.93 (74.70, 158.86)	Ref.
**Total Cholesterol**			
	≥12 months (N=27)	181.61 (161.01, 204.84)	0.40
	≥6-12 months (N=24)	176.44 (154.44, 201.56)	0.83
	<6 months (N=28)	174.47 (152.81, 199.20)	
**HDL Cholesterol**			
	≥12 months (N=27)	44.90 (37.17, 54.25)	**0.02**
	≥6-12 months (N=24)	41.34 (33.54, 50.95)	0.26
	<6 months (N=28)	37.79 (30.68, 46.53)	Ref.
**LDL Cholesterol**			
	≥12 months (N=27)	116.50 (97.94, 138.59)	0.37
	≥6-12 months (N=24)	108.24 (89.33, 131.15)	0.87
	<6 months (N=28)	109.58 (90.51, 132.67)	Ref.
**VLDL Cholesterol**			
	≥12 months (N=27)	16.14 (11.46, 22.73)	**0.03**
	≥6-12 months (N=24)	19.75 (13.52, 28.84)	0.50
	<6 months (N=28)	21.78 (14.94, 31.77)	Ref.
**Total to HDL Cholesterol Ratio**			
	≥12 months (N=27)	4.04 (3.35, 4.88)	0.08
	≥6-12 months (N=24)	4.27 (3.46, 5.26)	0.33
	<6 months (N=28)	4.62 (3.75, 5.68)	Ref.

Note: Model further adjusted for T2DM, pregnancy-related complications, including GDM, glucose intolerance, pre-eclampsia, hypertensive disorders of pregnancy, and depression, in addition to fasting status, demographic variables including maternal age, country of origin, marital status, annual household income, and education, maternal pre-pregnancy BMI and birth order. *Model estimates were back-transformed and presented as exponentiated marginal means for interpretability. †P values indicate the significance of comparison with breastfeeding <6 months as the reference group.

Supplemental Table S3. Regression coefficients (95% confidence interval) for the association of each month increase in breastfeeding duration with natural log-transformed 12-month postpartum lipids, excluding 27 participants who were currently breastfeeding at 12 months

Natural log-transformed lipids	Model 1		Model 2		Model 3		Model 4	
	Beta (95% CI)	P value	Beta (95% CI)	P value	Beta (95% CI)	P value	Beta (95% CI)	P value
Triglyceride	-0.02 (-0.08, 0.04)	0.56	-0.03 (-0.08, 0.03)	0.37	-0.04 (-0.11, 0.02)	0.16	-0.03 (-0.09, 0.03)	0.230
Total Cholesterol	0.01 (-0.01, 0.03)	0.32	0.01 (-0.01, 0.02)	0.42	0.00 (-0.02, 0.02)	0.70	0.01 (-0.01, 0.03)	0.491
HDL Cholesterol	**0.04 (0.01, 0.07)**	**0.01**	**0.04 (0.01, 0.07)**	**0.01**	**0.04 (0.01, 0.07)**	**0.01**	**0.04 (0.01, 0.07)**	**0.01**
LDL Cholesterol	0.00 (-0.03, 0.03)	0.94	0.00 (-0.03, 0.02)	0.87	0.00 (-0.03, 0.03)	0.79	0.00 (-0.03, 0.03)	0.99
VLDL Cholesterol	-0.02 (-0.08, 0.04)	0.56	-0.03 (-0.08, 0.03)	0.37	-0.04 (-0.11, 0.02)	0.16	-0.03 (-0.09, 0.03)	0.30
Total to HDL Cholesterol Ratio	-0.03 (-0.07, 0.00)	0.05	**-0.04 (-0.07, 0.00)**	**0.04**	**-0.04 (-0.07, 0.00)**	**0.03**	-0.03 (-0.07, 0.00)	0.06

Note: Model adjusted for fasting status, demographic variables including maternal age, country of origin, marital status, annual household income, education, maternal pre-pregnancy BMI, and birth order.

Supplemental Table S4. Comparison of MADRES population characteristics by breastfeeding duration categories

		<6 months N=220	≥6-11 months N=126	>=12 months N=142	F/χ^2	P
**Maternal age**		28.27 (5.57)	29.87 (6.67)	29.77 (5.94)	4.16	0.02
**Pre-Pregnancy BMI**		29.69 (6.96)	27.64 (7.23)	28.32 (5.15)	4.49	0.01
**Maternal country origin**						
	Latin America	78 (34.36)	60 (46.15)	73 (50.69)	11.02	0.03
	North America	129 (56.83)	59 (45.38)	61 (42.36)		
	Asia/Unknown	20 (8.81)	11 (8.46)	10 (6.94)		
**Maternal ethnicity**					3.35	0.19
	Non-Hispanic	45 (19.91)	32 (25.00)	23 (16.08)		
	Hispanic	181 (80.09)	96 (75.00)	120 (83.92)		
**Maternal marital status**					3.65	0.45
	Married	63 (27.75)	41 (31.54)	50 (34.72)		
	Cohabiting	86 (37.89)	49 (37.69)	57 (39.58)		
	Separated/Unknown	78 (34.36)	40 (30.77)	37 (25.69)		
**Household annual income**					2.70	0.26
	≥$30,000	45 (19.82)	31 (23.85)	39 (27.08)		
	<$30,000	182 (80.18)	99 (76.15)	105 (72.92)		
**Maternal education**					5.02	0.08
	Some college or above	88 (38.77)	65 (50.00)	68 (47.22)		
	High school or lower	139 (61.23)	65 (50.00)	76 (52.78)		
**Pre-Pregnancy BMI categories**					14.35	0.01
	Normal/Underweight	66 (29.07)	49 (37.69)	37 (25.69)		
	Overweight	61 (26.87)	44 (33.85)	58 (40.28)		
	Obese	100 (44.05)	37 (28.46)	49 (34.03)		
**Birth order**					4.34	0.11
	First	142 (62.56)	91 (70.00)	104 (72.22)		
	Second or later	85 (37.44)	39 (30.00)	40 (27.78)		
**Glucose intolerant/GDM/T2DM**					0.89	0.64
	No	142 (62.56)	84 (64.62)	97 (67.36)		
	Yes	85 (37.44)	46 (35.38)	47 (32.64)		
**Asthma**						
	No	186 (84.55)	99 (78.57)	121 (84.62)	2.39	0.30
	Yes	34 (15.45)	27 (21.43)	22 (15.38)		
**PE/HTN/PIH**					1.35	0.51
	No	178 (78.41)	104 (80.00)	120 (83.33)		
	Yes	49 (21.59)	26 (20.00)	24 (16.67)		
**Depression**						
	No	25 (11.01)	12 (9.23)	17 (11.81)	0.49	0.78
	Yes	202 (88.99)	118 (90.77)	127 (88.19)		

Supplemental Table S5. Adjusted lipids levels by breastfeeding duration and frequency*

	Triglycerides	P	Total-C	P	HDL-C	P	LDL-C	P	VLDL-C	P	T/H-C ratio	P
**Longitudinal breastfeeding frequency over 12months postpartum**												
0	78.50 (53.33, 115.54)	*Ref.*	166.45 (145.50, 190.42)	*Ref.*	35.91 (29.35, 43.93)	*Ref.*	110.91 (91.57, 134.34)	*Ref.*	15.70 (10.67, 23.11)	*Ref.*	4.64 (3.77, 5.70)	*Ref.*
1-6 times/day	103.34 (84.87, 125.82)	0.17	167.63 (156.53, 179.52)	0.92	40.28 (36.35, 44.64)	0.26	101.44 (92.01, 111.84)	0.36	20.67 (16.97, 25.16)	0.16	4.16 (3.74, 4.63)	0.31
≥7 times/day	98.82 (74.88, 130.40)	0.36	173.14 (157.21, 190.69)	0.65	45.77 (39.61, 52.89)	0.06	101.68 (88.62, 116.66)	0.48	19.76 (14.98, 26.08)	0.36	3.78 (3.26, 4.39)	0.13
**Breastfeeding duration**												
<6 months	120.51 (93.99, 154.50)	*Ref.*	168.47 (154.52, 183.69)	*Ref.*	40.10 (35.22, 45.64)	*Ref.*	99.09 (87.60, 112.07)	*Ref.*	24.10 (18.80, 30.90)	*Ref.*	4.20 (3.68, 4.80)	*Ref.*
6-11 months	90.23 (66.46, 122.50)	0.13	168.21 (151.23, 187.10)	0.98	40.29 (34.35, 47.26)	0.96	103.22 (88.70, 120.11)	0.66	18.04 (13.29, 24.50)	0.13	4.17 (3.54, 4.92)	0.95
≥12 months	73.72 (55.29, 98.31)	**0.02**	170.48 (154.23, 188.43)	0.87	40.98 (35.27, 47.61)	0.84	111.86 (96.99, 129.01)	0.24	14.74 (11.06, 19.66)	**0.02**	4.16 (3.56, 4.86)	0.93
P-interaction												
		0.28		0.14		0.16		0.07		0.28		**0.02**

Note: *Interaction product term of breastfeeding duration and frequency was NOT included in the model because it was not significant. Model adjusted for maternal age, country of origin, marital status, household annual income, education, pre-pregnancy BMI and birth order.
